# Feature preserving mesh network for semantic segmentation of retinal vasculature to support ophthalmic disease analysis

**DOI:** 10.3389/fmed.2022.1040562

**Published:** 2023-01-13

**Authors:** Syed Muhammad Ali Imran, Muhammad Waqas Saleem, Muhammad Talha Hameed, Abida Hussain, Rizwan Ali Naqvi, Seung Won Lee

**Affiliations:** ^1^Faculty of CS and IT, Superior University, Lahore, Pakistan; ^2^Department of Primary and Secondary Healthcare, Lahore, Pakistan; ^3^Department of Unmanned Vehicle Engineering, Sejong University, Seoul, Republic of Korea; ^4^School of Medicine, Sungkyunkwan University, Suwon, Republic of Korea

**Keywords:** ophthalmic diseases, retinal vasculature, retinal image segmentation, semantic segmentation, computer–aided diagnosis

## Abstract

**Introduction:**

Ophthalmic diseases are approaching an alarming count across the globe. Typically, ophthalmologists depend on manual methods for the analysis of different ophthalmic diseases such as glaucoma, Sickle cell retinopathy (SCR), diabetic retinopathy, and hypertensive retinopathy. All these manual assessments are not reliable, time-consuming, tedious, and prone to error. Therefore, automatic methods are desirable to replace conventional approaches. The accuracy of this segmentation of these vessels using automated approaches directly depends on the quality of fundus images. Retinal vessels are assumed as a potential biomarker for the diagnosis of many ophthalmic diseases. Mostly newly developed ophthalmic diseases contain minor changes in vasculature which is a critical job for the early detection and analysis of disease.

**Method:**

Several artificial intelligence-based methods suggested intelligent solutions for automated retinal vessel detection. However, existing methods exhibited significant limitations in segmentation performance, complexity, and computational efficiency. Specifically, most of the existing methods failed in detecting small vessels owing to vanishing gradient problems. To overcome the stated problems, an intelligence-based automated shallow network with high performance and low cost is designed named Feature Preserving Mesh Network (FPM-Net) for the accurate segmentation of retinal vessels. FPM-Net employs a feature-preserving block that preserves the spatial features and helps in maintaining a better segmentation performance. Similarly, FPM-Net architecture uses a series of feature concatenation that also boosts the overall segmentation performance. Finally, preserved features, low-level input image information, and up-sampled spatial features are aggregated at the final concatenation stage for improved pixel prediction accuracy. The technique is reliable since it performs better on the DRIVE database, CHASE-DB1 database, and STARE dataset.

**Results and discussion:**

Experimental outcomes confirm that FPM-Net outperforms state-of-the-art techniques with superior computational efficiency. In addition, presented results are achieved without using any preprocessing or postprocessing scheme. Our proposed method FPM-Net gives improvement results which can be observed with DRIVE datasets, it gives Se, Sp, and Acc as 0.8285, 0.98270, 0.92920, for CHASE-DB1 dataset 0.8219, 0.9840, 0.9728 and STARE datasets it produces 0.8618, 0.9819 and 0.9727 respectively. Which is a remarkable difference and enhancement as compared to the conventional methods using only 2.45 million trainable parameters.

## 1. Introduction

Ophthalmic diseases are increasing at an alarming rate. Early and automated diagnosis can help in preventing chronic ophthalmic disorders. Ophthalmic diseases include glaucoma, macular degeneration, Sickle cell retinopathy (SCR), and hypertensive and diabetic retinopathy. All of these are common but serious ophthalmic diseases and can lead to vision loss if not diagnosed at an early stage. An ophthalmological image assessment is commonly used for retinal disease analysis which shows retinal vessel changes that can lead to vision loss problems (1). Another vision loss syndrome that is affected by retinal ischemia is Sickle cell retinopathy (SCR). Reduced vessel density and altered vasculature shape are symptoms of sickle cell retinopathy (SCR) illness. Important biomarkers for early SCR identification include retinal vessels (1). A high blood sugar level causes the retinal illness known as diabetic retinopathy, which causes retinal vessels to enlarge or leak (2). A retinal condition called hypertensive retinopathy causes restricted retinal vessels as a result of elevated blood pressure which can be especially noticeable in the micro-vasculature (3). The location of the retinal vascular blockage can be determined using retinal vascular changes, which are often seen in bigger arteries. These retinal vascular illnesses are strongly related to the retinal morphologies of arteries and some other vessel diseases (1). Aimed at the early finding of chronic ophthalmic disorders by using different fundus images are retinal vessels which are a vital biomarker.

Precise retinal image analysis is necessary for early ophthalmic diagnosis. The complicated nature of the retinal blood vessels makes them essential biomarkers for diagnosing and analyzing many retinal disorders. However, it can be difficult to detect little changes in retinal vessels. Retinal vascular morphology includes location, thickness, tortuosity, formation, and removal, and is linked to several ocular illnesses (4). Ophthalmologists assess and record changes in the retinal vasculature manually. This procedure is time-consuming and labor-intensive. Additionally, the diagnosis of the aforementioned disorders can be made using the size of the retinal vessels, which is a distinct change that is difficult to find and evaluate using manual image analysis (4) by medical practitioners. Automatic illness inquiry is becoming more prevalent as deep learning technology progresses to help doctors make quicker and more accurate diagnoses (1). As the analysis of medical images is a crucial component of computer-aided disease diagnosis. Due to their dependability and adaptability, artificially intelligence-based approaches are more well-known in syndrome investigation than traditional image processing techniques. Deep learning-based algorithms help medical specialists to analyze various diseases using computer vision approaches (1–8).

Computer vision has an immense potential to evaluate these retinal disorders through image analysis for premature diagnosis. Ophthalmologists and other medical professionals are dealing with a variety of diagnostic challenges with the use of deep learning techniques like medical image segmentation. Semantic segmentation using deep learning is a cutting-edge technology for medical image segmentation that helps to avoid the manual processing of images for disease or symptom diagnosis (7). Most of the work done already for the retinal vessels segmentation is based on general image processing schemes; in which several image augmentation patterns were used to enhance the image contrast and detection process, which is usually based on some specific threshold. In such a case, a specific threshold cannot perform better with changes in the image acquisition system. Therefore, to incorporate the portability of the method, learning-based-segmentation algorithms are famous.

The process of semantic segmentation entails giving class labeling to each pixel of the image. Semantic segmentation may be thought of as the process of identifying an image class and isolating it from the other image classes by overlaying a segmentation mask on top of it. Features extraction features and representations are frequently necessary for semantic segmentation to obtain an optimal correlation of the image, effectively reducing the noise. The suggested study explains the deep-learning-based semantic segmentation technique called Feature Preserving Mesh Network (FPM-Net) for the detection of precise retinal vasculature in fundus images. Here, we use multiple convolution layers with a combination of depth-wise separable convolutions to lessen the overall trainable parameters. Due to the spatial information being lost as a result of the pooling of layers, we employed feature-preserving blocks to maintain feature map sizes that were large enough to handle the lost spatial information. The dense connection prevents the vanishing gradient issue that plagues traditional networks' feature latency (9), leading to improved training. This feature-preserving block results in enhanced sensitivity of the suggested FPM Network without using costly preprocessing techniques. Finally, preserved features, low-level input image information, and up-sampled spatial features are aggregated at the final concatenation stage for improved prediction accuracy.

The suggested FPM-Net method was applied to the fundus images in three different publically available databases (5), The technique is reliable since it performs better even after being trained on the DRIVE database (2), STARE database (10), and CHASE-DB1 (10), making it appropriate for images captured under various situations without retraining. After experiments, the outcomes of segmentation concluded a meliorated performance with accuracy (Acc), sensitivity (Se), specificity (SP), and area under the curve (AUC) for retinal vasculature segmentation. The suggested method FPM-Net has a much better performance than conventional methods.

The structure of this paper is as follows. Some conventional and automated methods relevant to this work will be presented in Section 2. The embedding strategy and method are given in Section 3. Results can be found in Section 4 and discussions in Section 5. In Section 6, a conclusion is provided.

### 1.1. Research motivation

An increasing rate of growth is being observed in ophthalmic illnesses. Chronic ocular problems can be avoided with early and automated diagnosis. Retinal vascular alterations, which are frequently observed in larger arteries, can be used to pinpoint the exact location of the retinal vascular occlusion. The retinal morphology of arteries and a few other vessel diseases are closely related to these retinal vascular diseases (1). Retinal vessels, an important biomarker, are used to detect chronic retinal problems early by employing various fundus image observations. However, it could be challenging to spot slight variations in retinal vessels. The location, thickness, tortuosity, creation, and removal of retinal vessels all affect their morphology and are associated with several retinal diseases (4). Ophthalmologists manually evaluate and document changes to the retinal vasculature. This process takes a lot of time and effort. Additionally, the size of the retinal vessels, which is a unique alteration that is challenging to discover and analyze using manual image analysis (4), can be used to diagnose the aforementioned illnesses.

The evaluation of these retinal illnesses by image processing for early diagnosis has enormous potential for computer vision. Ophthalmologists and other medical practitioners are using deep learning methods like medical image segmentation to address a range of diagnostic issues. Deep learning-based semantic segmentation is an absolute technique for medical image segmentation that eliminates the need for manual image processing for the identification of illness or symptom (7).

## 2. Related work

Automated approaches are important for lowering the diagnostic workload of medical specialists, and the detection of retinal vasculature can be helpful for the premature investigation of a variety of eye-related diseases. There are two basic methods for segmenting retinal vessels: feature-based deep learning techniques and traditional image processing approaches. Various studies have been conducted using traditional techniques and common image-processing algorithms. Here we describe recent advances in image analysis and deep functionality learning techniques. Traditional image processing techniques have been studied recently, and deep learning-based techniques have grown with great constancy and performance (1). Researchers have previously developed a variety of machine-learning methods to separate the blood vessels from imaging the retinal fundus. When handling testing conditions such as recognized low-contrast micro-vessels, vessels with focal reflexes, and vessels within the sight of diseases, a significant number of visible retinal vessel division techniques are prone to more unfavorable results (2).

Numerous image-enhancement techniques are frequently used before thresholding in traditional image processing-based vessel segmentation approaches. In addition to using contrast-limited adaptive histogram equalization (CLAHE) to rise the divergence of fundus images, Alhussein et al. developed a segmentation method centered on Wiener and morphological filtering (3). The primary vascular region was located using the detector-based vessel identification approach developed by Zhou et al., and after the noise was removed, a Markov model was used to locate retinal vasculatures (11). In a similar vein, Ahamed et al. reported segmenting the autonomic vasculature multiscale line detection-based approach. To increase contrast, they added CLAHE toward the green channel and for the final segmentation, they combined morphological thresholding and hysteresis (4). For the segmentation of retinal vessels, Shah et al. employed a multiscale line-detection technique. The images aimed at vessel segmentation were made better on the green channel using Gabor wavelet superposition and multiscale line detection (4). Using top hat with homomorphic filtering, Soto et al. presented a three-stage method. Following the initial stage of visual smoothing for image enhancement, two phases were employed to separately segment both thin and thick vessels. The segmentation findings were improved in the final stage with the application of morphological post-processing (5). Li et al. introduced an unsupervised technique in which integrated-tube marked point processes were applied to extract the vascular network from the images and to preprocess the images, image-enhancing techniques were applied. Utilizing the discovered tube width expansion, the final segmentation was carried out (7). Aswini et al. introduced an un-supervised technique consisting of hysteresis thresholding with two folds to identify retinal vessels. In their approach, morphological smoothness and background reduction were used to improve the fundus images before thresholding (8). Another approach based on image processing segmented the vasculature using the curvelet transform and line operation after pre-processing using anisotropic diffusion filtering, adaptive histogram equalization, and color space translation (11). Sundaram et al. suggested a hybrid strategy based on bottom-hat transform and multiscale image augmentation, where the segmentation work was carried out using morphological procedures (10). To reduce the aggravating noise that prevents vessel segmentation, using a probabilistic patch-based denoiser was recommended by Khawaja et al. (2) that combines a customized Frangi filter with a denoiser. After the CLAHE procedure, images are enhanced using an aggregated block-matching 3-D speckled filter, Naveed et al. suggested an unsupervised technique. Multiscale line detectors along with Frangi detectors were used in their model to segment data (12).

All the above-discussed methods are traditional image processing and some deep-feature-based learning techniques are used to investigate retinal vasculature segmentation. Learning-based approaches are increasingly well-known because, through feature-based learning, they may imitate the expertise of medical professionals. Furthermore, techniques for image augmentation make it possible to complete the task with lesser training samples. For supervised vessel segmentation, Oliveira et al. suggested an entirely convolutional deep-learning technique. They employed a multiscale convolutional network in a patch-based scenario, which was investigated by some kind of stationary wavelet transform (13).

Fraz et al. integrated the vessel centerlines identification method with the morphological bit plane slicing technique. They coupled bit plane slicing with vessel centerline on the enhanced gray-level images of retinal blood vessels (14). In addition to performing a mathematical morphological procedure on the image, Ghoshal et al. suggested an enhanced vascular extraction method from retinal images. They made negative grayscale images from the original and the image that had been removed from the vessels, then they excised to balance the image and then improved to produce thin vessels by turning the produced image into a binary image. To produce the vessel-extracted image, they finally combined the thin vessel image and binary image. They claimed that their performance results were satisfactory (15). The answers from the two-dimensional Gabor wavelet transform at various scales of each pixel were utilized as features by Soares et al. after they used this transform with supervised learning. They rapidly categorized a complicated model using a Bayesian classifier (16). To determine the properties necessary for segmenting retinal blood vessels, Ricci and Perfetti suggested a technique based on line operators. Because their model uses a line detector to analyze the green channel of retinal images, it is quicker and requires fewer features than prior approaches (17). A multi-layered forward-oriented artificial neural network was trained using the suggested artificial neural network approach by Marin et al. using a seven-dimensional feature vector. They employed the sigmoid activation function in each neuron of the three-layer network. They claimed that additional datasets are also successfully used by the trained network (18). A technique using a CNN architecture was created by Melinscak et al. to determine if each pixel is a vessel or a backdrop (19). According to Wang et al. proposal for a new retinal vascular segmentation approach that uses patch-based learning and Dense U-net, the approach seems attractive in terms of standard performance criteria (20). For segmenting retinal vessels, Guo et al. developed a CNN-based two-class classifier comprising two convolution layers and pooling layers, one dropout layer, and one loss layer. They concluded that the suggested approach had good accuracy and was quick to teach (21). Concerning the information loss brought on by image scaling during preprocessing, Leopold et al. proposed PixelBNN, an effective deep learning system for automatically segmenting fundus morphologies, and reported that it had a reduced test time and reasonably high performance (9). Technology advancements have produced images with a higher pixel density, sharp features, and a lot of data. As a result, good image quality can satisfy the requirements for actual application in image analysis and image comprehension (22). CNN is effective in classifying images and detecting objects, although the results vary depending on the network design, activation function chosen, and input picture quality. Poor quality input images have a detrimental impact on a CNN's performance, according to research (23), even if it is not immediately apparent. IterNet, a novel model based on UNet that can uncover hidden vessel information from the segmented vessel image rather than the raw input image, was proposed by Li et al. IterNet is made up of several mini-UNet iterations that can be up to four times deeper than a typical UNet (24). A new approach for segmenting blood vessels in retinal images was put out by Tchinda et al. The artificial neural networks and conventional edge detection filters are the foundation of this approach. The features vector is first extracted using edge detection filters. An artificial neural network is trained using the obtained characteristics to determine whether or not each pixel is a part of a blood artery (25).

According to the properties of the retinal vessels in fundus images, a residual convolution neural network-based retinal vessel segmentation technique is presented. The encoder-decoder network structure is built by joining the low-level and high-level feature graphs, and atrous convolution is added to the pyramid pooling. The improved residual attention module and deep supervision module are used. The results of the trials performed using the fundus image data set from DRIVE and STARE demonstrate that this algorithm can successfully segment all retinal vessels and identify related vessel stems and terminals. This approach can identify more capillaries and is viable and successful for segmenting retinal vessels in fundus images (11). One of the most serious infectious diseases in the world, tuberculosis causes 25% of all preventable deaths in underdeveloped nations. This cross-sectional descriptive research set out to assess the effects of ocular TB on visual acuity both before and after 2 months of vigorous anti-tubercular treatment. Three individuals with pleural TB, seven with disseminated tuberculosis, and 133 with pulmonary tuberculosis comprised the sample. Every patient got a standard eye examination, which included measuring visual acuity and performing necessary indirect ophthalmoscopes, biomicroscopy, applanation tonometry, and fluorescence angiography. None of the patients exhibited tuberculosis-related vision impairment. The incidence of ocular involvement was determined to be 4.2% (6/143). Five of the six individuals with ocular involvement and one of the suspected ocular lesions satisfied the diagnostic criteria for probable ocular lesions. Two individuals showed bilateral findings of different ocular lesions: one had sclera uveitis and the other had choroidal nodules. The remaining four patients all had unilateral lesions, including unilateral choroidal nodules in the right eye, unilateral choroidal nodules in the left eye, and unilateral peripheral retinal artery blockage in the right eye (two cases). After 2 months of rigorous therapy, patients made favorable improvements with no discernible visual loss (26).

## 3. Suggested methodology

### 3.1. Suggested FPM-Net's outline

**Figure 1:**
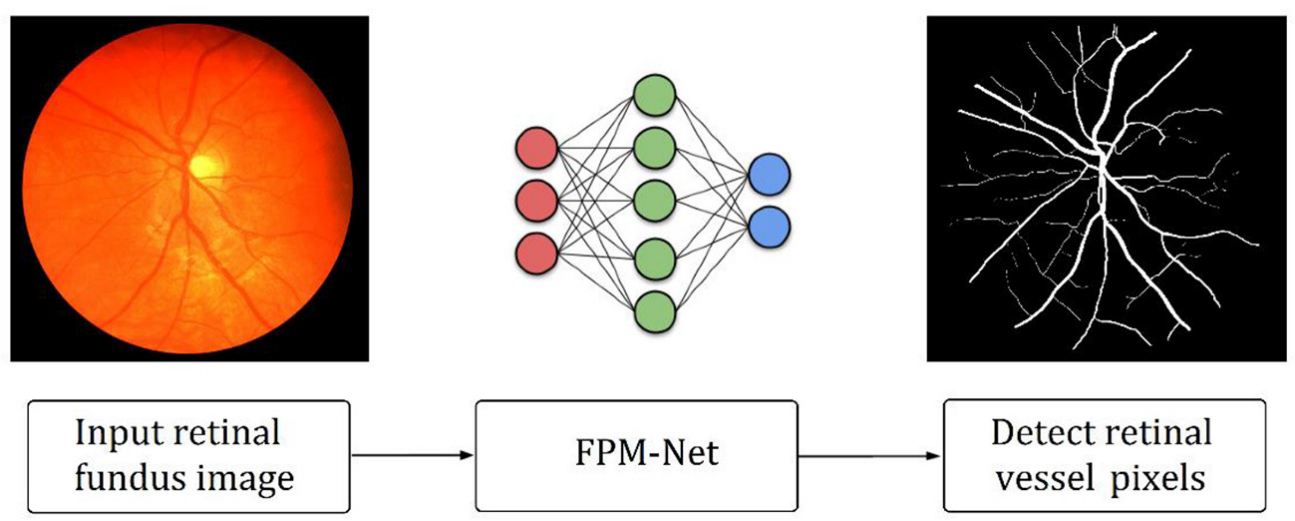
Outline of the suggested FPM-Net approach.

As explained in section 2, retinal vessels are assumed as an important potential biomarker for the diagnosis of many ophthalmic diseases. A very growing number of ophthalmic illnesses are found in a large number of people around the globe. Preventing persistent ocular problems can be aided by early and automated diagnosis. Precise retinal image analysis is necessary for early ophthalmic diagnosis. Numerous AI-based techniques provide intelligent solutions for automatic retinal vessel recognition. However, segmentation performance, complexities, and computing efficiency were significantly constrained by previous approaches. Due to the vanishing gradient issue, and conventional architectural design, the majority of the currently used approaches specifically failed to achieve a higher true positive rate. Figure 1 provides an outline of the suggested technique. The suggested technique simply uses fundus images as input deprived of applying the requirement of any pre-processing scheme. FPM-Net is applied to the input image for pixel-wise classification. The suggested network categorizes each pixel into two major categories: “vessel” (for vessel pixel) and “background” (for pixels other than vessels). Because of this, it provides a binary segmentation mask with values of “1” on vessels as well as “0” on the other classes. FPM-Net incorporates a feature-preserving block for enhanced performance and fast convergence.

### 3.2. Architecture of suggested FPM-Net

**Figure 2:**
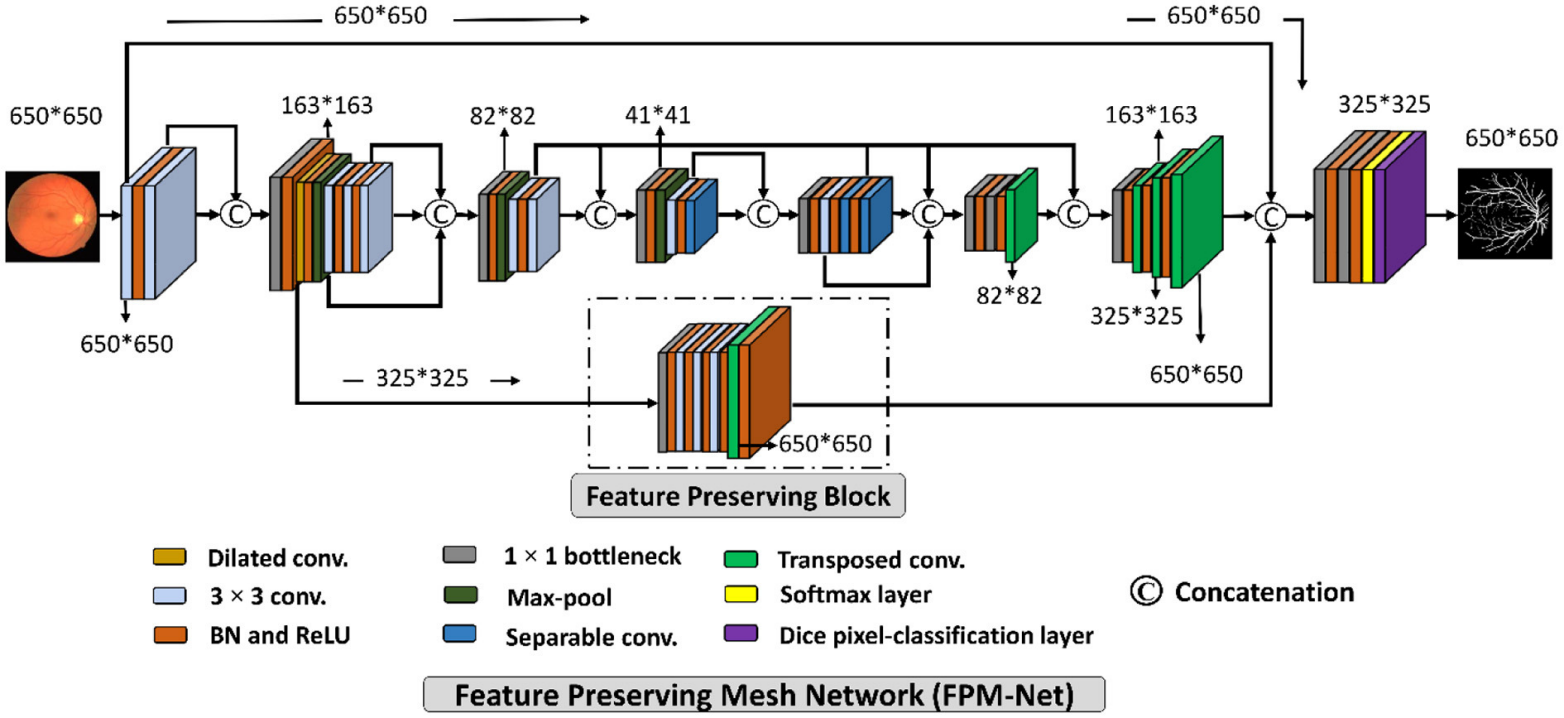
The suggested Feature Preserving Mesh Network (FPM-Net) architecture for retinal vessels segmentation.

A suggested network for segmenting vessels that was created especially to improve the sensitivity (a better true positive rate) of retinal vascular detection is called a Feature Preserving Mesh Network (FPM-Net). The suggested FPM-Net is shown in Figure 2. Observe (Figure 2) that FPM-Net is a dense network composed of multiple convolution operations, and a shallow feature up-sampling block (FUB) followed by mesh-connected dense feature down-sampling block (FDB), and this overall architecture differs from conventional semantic segmentation networks like Seg-Net, U-Net, and DeepLabV3 in terms of encoder-decoder architecture where the decoder is same as an encoder.

To address above mentioned issues with conventional networks, FPM-Net is following four design principles. First, multiple uses of convolution layers in deep networks (e.g., VGG16) cause spatial loss if they are used without a feature reuse policy and the overall performance deteriorates (27). Following Dense-Net (22), to cover the spatial loss, dense connections are used between the convolution layers available in the network which guarantees the immediate feature transfer without latency. Secondly, the convolution layers with a larger number of channels contribute to increasing the number of learnable parameters substantially. To reduce the network cost, we use depth-wise separable convolution on the deep side of the network. Third, the spatial information that is available in the initial layers is very important as it contains the low-level features to represent the edges. The FPM-Net is utilizing a dense mesh that is connecting all the convolutional layers and transfers this valuable low-level information from FDB to FUB directly. This ensures the immediate edge information transfer without latency which results in better segmentation performance and quicker convergence of the network. Fourth, the multiple pooling operation causes severe spatial information loss that inevitably leads to a deterioration in performance (28). Traditional convolutional neural networks employ excessive pooling operations for reducing the feature map size which is equally important to control memory usage. To cover the issues created by multiple pooling layers (minor information loss due to small feature map size), FPM-Net is using the feature preserving block (FPB) which keeps the feature map size larger to represent approximately all the valued features that can signify the vessel pixels. FPB is composed of a few low-cost convolution layers, and it is responsible to transfer a large feature map to the FUB. This FPM-Net provides better segmentation accuracy and is computationally efficient because it does not require a huge number of parameters for its training. This structure is completely diverse from traditional structures like Segmentation Networks (SegNet) (29) and U-Shaped Network (U-Net) (30), which employ a decoder similar to an encoder to produce an architecture that is excessively deep and has a lot of trainable parameters with many channels. Figure 2 explains the connectivity pattern of FPM-Net.

**Figure 3:**
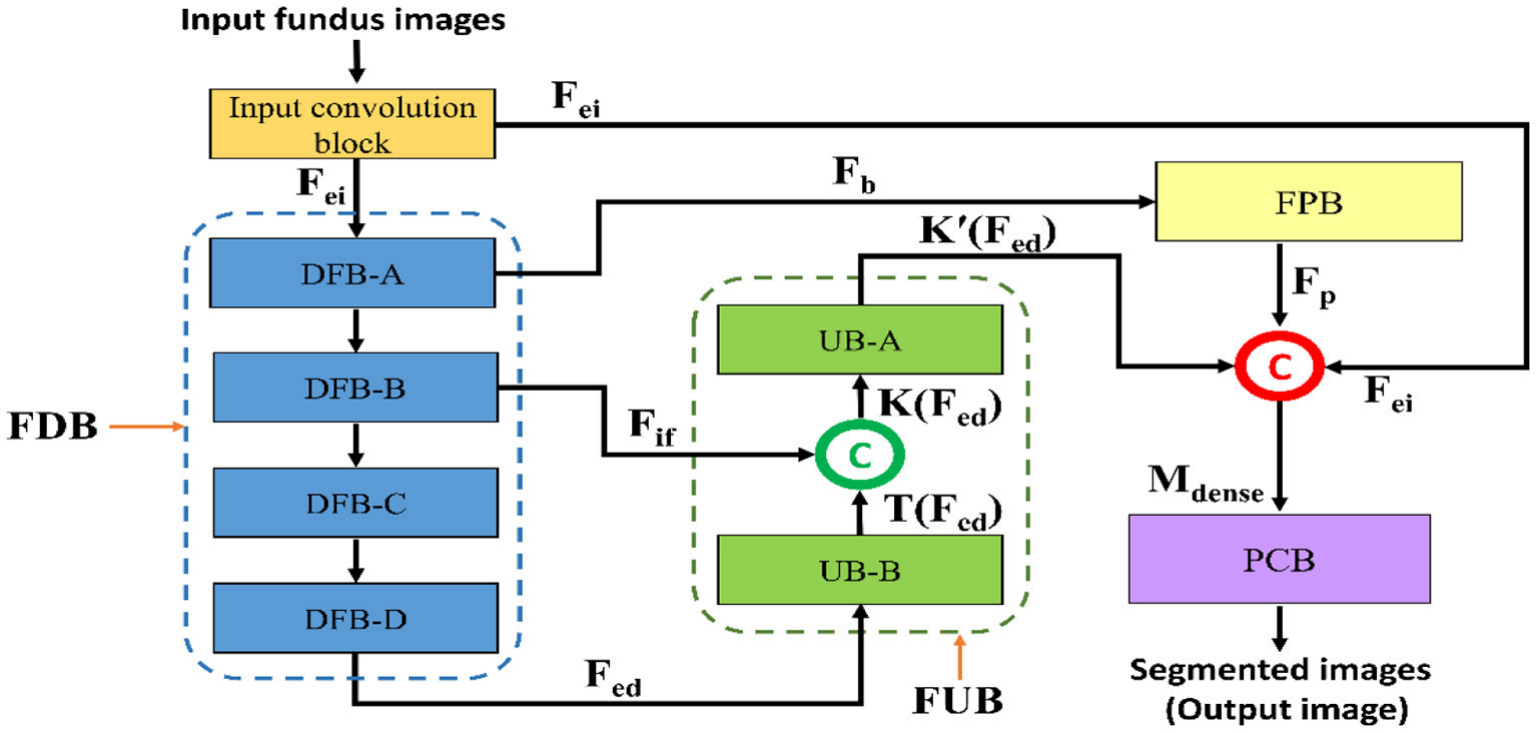
The schematic diagram for FPM-Net connectivity, FDB, FUB, FPB, and PCB represents the feature down-sampling block, feature up-sampling block, feature preserving block, and pixel classification block, respectively.

Figure 3 represents a schematic diagram for FPM-Net interconnection and the solid feature concatenation standards. The input convolution block uses the fundus images as input, runs them through many convolutional layers in FDB to extract significant features F_ed_ for the investigation of the retinal vasculatures, and then sends the enhanced dense features F_ed_ to the UB-A of FUB. K (F_ed_) is created by concatenating the enhanced dense features T(F_ed_) and intermediate feature information F_if_ that were acquired by the DFB-B and DFB-D, respectively. The K(F_ed_) feature, represented by Equation (1), is produced *via* depth-wise concatenation using both T(F_ed_) and F_if_, where © represented depth-wise concatenation in green color. The F_b_ feature is being added to the feature-preserving block from the DFB-A. Since there haven't been any significant pooling operations, the feature F_p_ originating from the feature-preserving block (FPB) contains rich feature information that corresponds to the majority of the vessels in the images transfer to the final concatenation represented in red color.


(1) $$\text{K}({\text{F}}_{\text{ed}})=\text{T}({\text{F}}_{\text{ed}}){\text{©F}}_{\text{ei}}$$

(2) $${\text{M}}_{\text{dense}}=\text{K′}({\text{F}}_{\text{ed}}){\text{©F}}_{\text{p}}{\text{©F}}_{\text{if}}$$

Here, M is a densely concatenated feature made through the K'(F_ed_), a feature after the up-sampling block, F_p_ preserved features, upcoming from the feature preserving block, and edge information f_ei_, upcoming from the input convolution block. Where © denotes depth-wise concatenation. After final concatenation represented in red color concluded the output result having Equation (2).

### 3.3. Structure of feature preserving block

**Figure 4:**
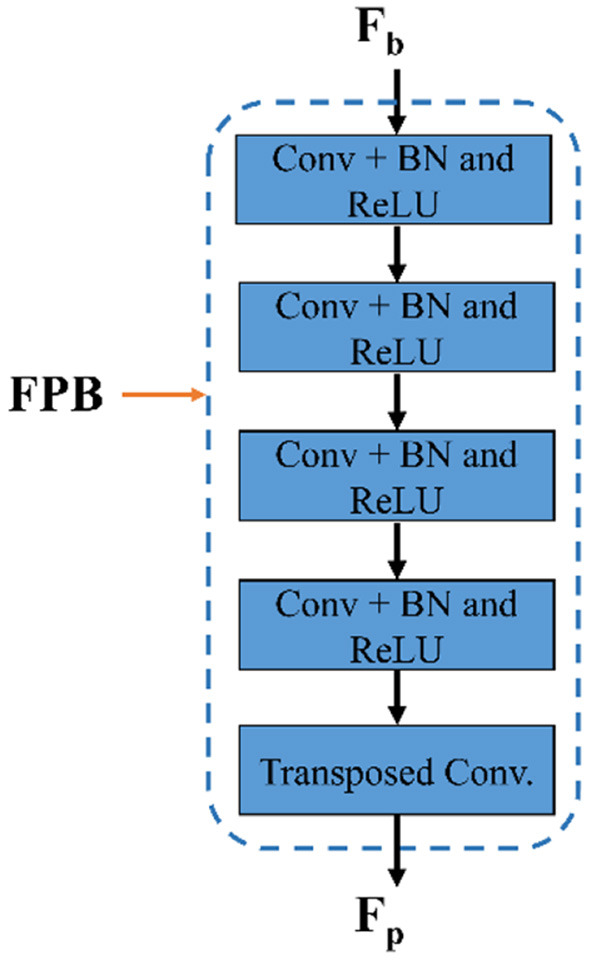
The structure of feature preserving block.

As shown in Figure 2, the suggested FPM-Net uses a feature-preserving block (FPB) to preserve valuable spatial information and disseminates it for the final concatenation. FPB takes the input from the dilated convolution, performs its function, and provides the feature results for the concatenation to the later layer. Because in the initial layer there is potential spatial information and features that can signify most of the vasculatures which will be helpful in the final prediction. The main problem that occurs while segmenting the image, the small objects were lost called the vanishing gradient but in FPB this vanishing gradient issue is solved. It simply uses three convolution layers and one transposed convolution to increase feature map size the feature map is resized to its original size using transposed convolution. As discussed above, the edge information from the initial layer, preserved features from FPB, and enhanced dense features are concatenated in the final stage which will boost the segmentation performance and improve the overall accuracy. After the final concatenation, softmax and pixel classification layers are utilized. The schematic structure of the feature-preserving block is mentioned in Figure 4.

### 3.4. Structure of suggested pixel classification block

The final concatenation has shown in red before the pixel classification block is given the rich features, K, from the up-sampling block. The PCB encompasses a 1 × 1 bottleneck (used to reduce the number of channels for pixel classification block), softmax, and dice pixel classification layer. The image pixels are categorized using a dice pixel classification layer that uses dice loss to solve the class imbalance and give improved segmentation. In this instance, “vessel” and “background” are two segmentation classes with values of “1” and “0,” respectively. The pixel classification block is made up of a convolution whose filters are matched to the number of classes. The image pixels are identified using a pixel classification layer that uses dice loss to solve the class imbalance (31) and give improved segmentation. The dice loss (L_DL_) is represented mathematically as,


(3) $$\begin{array}{l} \text{LDL}=1-\left(\frac{2\times \sum_{\text{i}}^{\text{j}}{\text{Q}}_{\text{p}-\text{i }}{\text{R}}_{\text{T}-\text{i}}}{\sum_{\text{i}}^{\text{j}}{\text{Q}}_{\text{p}-\text{i}}^{2}\;+\;{\text{R}}_{\text{T}-\text{i}}^{2}}\right) \end{array}$$

Where j refers to all of the image's observable pixels, i is the pixel under consideration, Q refers to the predicted labels, and R refers to the actual ground truth labels. R_T − i_ is the actual ground truth label, and Q_p − i_ is the expected possibility that pixel i belongs to a certain class.

## 4. Experimental results

### 4.1. Datasets

Intend to find results, vessels analysis was done on the DRIVE (2), CHASE-DB1 (10), and STARE (10) datasets for the suggested technique and additional studies for overall evaluation. These datasets are publicly accessible, and pixel-wise expert annotations on the photographs allow researchers to assess the algorithms. The following describes these datasets.

**Figure 5:**
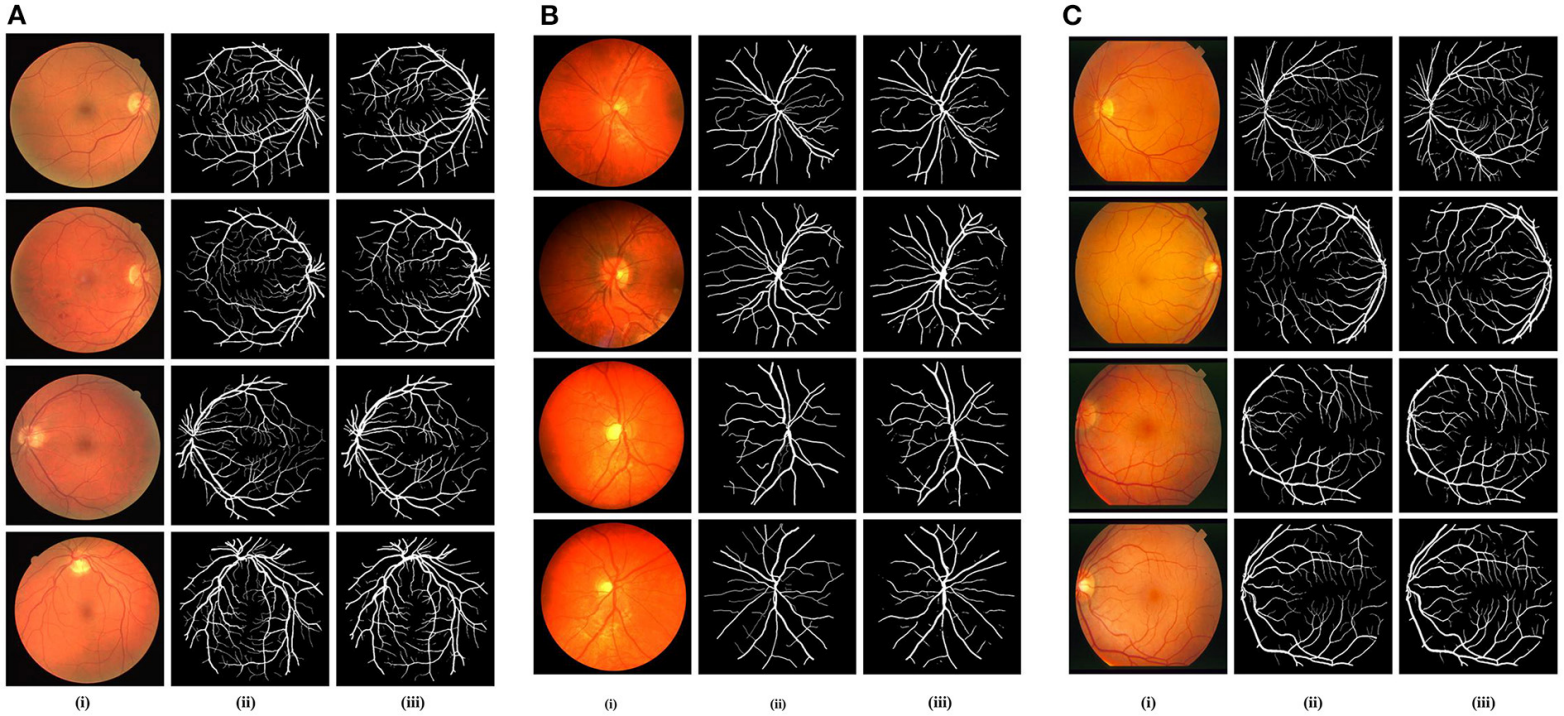
**(A)** DRIVE Dataset visualizations of the suggested FPM-Net: (i) Input original image, (ii) Expert annotation (Ground truth), and (iii) Predicted mask by FPM-Net. **(B)** CHASE-DB1 Dataset visualizations of the suggested FPM-Net (i) Input original image, (ii) Expert annotation (Ground truth), and (iii) Predicted mask by FPM-Net. **(C)** STARE Dataset visualizations of the suggested FPM-Net (i) Input original image, (ii) Expert annotation (Ground truth), and (iii) Predicted mask by FPM-Net.

In the DRIVE dataset, 40 red, green, and blue fundus images in total are included in the collection. The dataset comes with carefully separated ground truths for analysis. The images have a 565 x 584-pixel resolution and a 45° field of view (FOV). For improved training, the 20 training images are enhanced. Examples of expertly annotated images on or after the DRIVE dataset are displayed in Figure 5A. In the CHASE-DB1 dataset with 28 images using a fundus camera (Nidek NM-200D) with a typical FOV of 30°. Complying with the validation requirement, with a total of 28 images, 20 images (with augmentation) were used in our studies for training purposes and the remaining eight for testing purposes. Examples of image pairings with professional annotations are shown in Figure 5B. The STARE dataset is a collection of 20 retinal images taken by a TopCon TRV-50 with a FOV of 35°. For assessment reasons, professional image annotations are given per image. We used cross-validation using the leave-one-out method in our studies, in which training is done on 19 images and just one left for testing. Similarly to this, each image in the 20 studies was chosen specifically for testing. Twenty experiments on average were used to get the data. Examples of image pairings with professional annotations from the STARE dataset are shown in Figure 5C. The training and testing image descriptions for each dataset are displayed in Table 1.

**Table 1:** Details of the testing and specifications of all three used datasets in our method.

**Name of dataset**	**Total images**	**Images division (training, testing)**	**Experimentation**
DRIVE (2)	40 images	20, 20	One experiment
CHASE-DB1 (10)	28 images	20, 8	One experiment
STARE (10)	20 images	19, 1	20 experiments

### 4.2. Experimental environment and augmented data

The suggested FPM-Net was developed using Microsoft Windows 10, MathWorks MATLAB R2022a, with a laptop having specifications. An Intel Core i7-11800H processor and RAM of 16 GB. The tests were performed using an NVIDIA GeForce RTX 3070 8GB GDDR6 graphics processing unit. Without using any method for weight initialization, migration, sharing, or fine-tuning from previous networks, the suggested models were trained from scratch. **Tables 3A–C** lists the important training hyperparameters.

Deep learning's segmentation effectiveness is closely correlated with the capacity of training data with labels; effective training requirements, and a substantial amount of training data with labels. To boost the quantity of data, we used image flipping and translation. The modified augmentation method involved flipping 20 original images in both vertical direction and horizontal directions to produce a total of 60 images. Then, the total images produced after the flipping procedure are 3,000, from the DRIVE dataset were produced by repeatedly translating these 60 images into (x, y) values and then continuing to flip them. A training set is prepared using a random image generation procedure, where the points (x, y) satisfy the conditions. The CHASE-DB and STARE databases were similarly enhanced to provide 1,500 and 1,300 images, respectively.

Considering the training details FPM-Net utilized an epsilon of 0.000001, and the initial learning rate of 0.00005 was applied. Global L2 normalization is utilized for training due to the benefits of quicker convergence and robustness over rising variation. To train the FPM-Net, a mini-batch size of 16 images is used because it is a dense network and requires less GPU memory due to bottleneck layers. In 25 epochs, both networks converge (5,000 iterations).

### 4.3. Ablation study for the suggested FPM-Net

The rich edge information is found in the starting layers by the network detection. By minimizing the vanishing gradient problem, the network's convergence is aided by the import of this data through skip connections (44). To investigate the efficacy of preserved features and dense connectivity for the suggested FPM-Net, an ablation study was conducted. In the ablation study, the training was done on FPM-Net architecture with and without FPB. Table 2 shows that, while maintaining the almost same number of parameters, FPB with preserved feature outperformed FPM-Net with dense connectivity in terms of true positive rate (SE), with a greater true positive rate. Table 2 clearly shows that feature concatenation caused a significant performance difference.

**Table 2:** Performance measures with ablation study.

**Method**	**SE**	**SP**	**Acc**	**AUC**	**Parameters**
FPM-Net (without FPB)	0.8035	0.9801	0.9591	0.9790	2.44M
FPM-Net (with FPB)	0.8285	0.9827	0.9692	0.9851	2.45M

FPM-Net, Feature preserving mesh network; FPB, Feature preserving block; SE, sensitivity; SP, specificity; Acc, Accuracy; AUC, Area under Curve; ms, microseconds.

### 4.4. Evaluation of suggested network

For the suggested network output, FPM-Net offers a mask that displays all of the background and vessel pixels as “0” and “1,” respectively. Sensitivity (SE), Specificity (SP), Accuracy (Acc), and area under curve AUC, to measure the performance of segmentation which are frequently utilized to assess how well-retinal images are segmented, were computed using the output mask of the suggested network and expert annotations (16). SE is denoted as a true positive rate, which illustrates how well the network can find vessel pixels. The SP as a true negative rate demonstrates the capacity to identify non-vessel pixels. The whole percentage of accurate predictions made thru the approach is represented by Acc. Equations (4)–(6) give the respective expressions for SE, SP, and Acc. A pixel with the prefix TP is identified in the expert's annotation as a vessel pixel and is projected to be one. FN denotes a pixel that the expert annotation classifies as a vessel pixel even if it is expected to be a background pixel. A pixel with the prefix TN is identified in the expert's annotation as a vessel pixel and is expected to be one. FP denotes a pixel that the expert annotation classifies as a background pixel but which is expected to be a vessel pixel.


(4) $$\begin{array}{l} SN=\frac{TP}{TP\;+\;FN} \end{array}$$

(5) $$\begin{array}{l} SP=\frac{TN}{TN\;+\;FP} \end{array}$$

(6) $$\begin{array}{l} Acc=\frac{TP\;+\;TN}{TP\;+\;FN\;+\;FP\;+\;TN} \end{array}$$

### 4.5. Comparison with other conventional techniques

To evaluate and compare the suggested FPM network with the conventional techniques, vessel analysis was done on the publicly accessible DRIVE CHASE-DB1, and STARE datasets. For the vessel category and the background category, the network generates a mask with both the corresponding grayscale values of “1” and “0,” respectively. The visual outcomes of the suggested strategy for the three datasets stated above are shown in Figure 5. The suggested FPM-Net network's segmented image with the mask overlapped is shown in the figures along with the original images that were used as input into the network, experts provided the expert annotated image to evaluate research methods, the predicted mask at the network's production, and the predicted mask itself. The Numerical Comparison of Suggested FPM-Net utilizing the most recent technique is described in Tables 3A–C. By using our proposed method FPM-Net, there is significant improvement can be observed with DRIVE datasets, it gives S_e_, S_p_, and A_cc_ as 0.8285, 0.98270, 0.92920, for CHASE-DB1 dataset 0.8219, 0.9840, 0.9728 and STARE datasets it produces 0.8618, 0.9819 and 0.9727 respectively. Which is a remarkable difference and enhancement as compared to old and conventional methods.

**Table 3A:** The comparison of the DRIVE data set's segmentation results using various segmentation techniques.

**Method**	**Year**	**S_e_**	**S_p_**	**A_cc_**
Cross modality learning (17)	2015	0.7569	0.9816	0.9527
GMM classifier (18)	2015	0.7249	0.9830	0.9620
SP model (19)	2016	0.7811	0.9807	0.9535
CRF model (20)	2016	0.7897	0.9684	–
VS method (21)	2017	0.7779	0.9780	0.9521
RU-Net and R2U-Net (9)	2018	0.7792	0.9813	0.9556
LadderNet (22)	2018	0.7856	0.9810	0.9561
U-Net+joint losses (23)	2018	0.7653	0.9818	0.9542
CTF-Net (24)	2018	0.7979	0.9857	0.9685
Three-stage DL Model (25)	2019	0.7631	0.9820	0.9538
SD-Unet (32)	2019	0.7891	0.9848	0.9674
Dilated Conv. (33)	2019	0.7903	0.9813	0.9567
GFM (15)	2020	0.7614	0.9837	0.9604
DL methods (10)	2020	0.7979	0.9794	0.9563
AA-UNet (34)	2020	0.7941	0.9798	0.9558
EDC-Net (35)	2020	0.7092	0.9820	0.9447
Iternet (36)	2020	0.7735	0.9838	0.9673
MLC scheme (37)	2021	0.7761	0.9792	0.9519
LAC network (38)	2021	0.7921	0.9810	0.9568
ResDo-UNet (39)	2021	0.7985	0.9791	0.9561
FPM-Net (proposed)	2022	0.8285	0.98270	0.96920

“–” means the value is not available in the relevant research study.

**Table 3B:** The comparison of the CHASE-DB1 data set's segmentation results using various segmentation techniques.

**Method**	**Year**	**S_e_**	**S_p_**	**A_cc_**
U-Net (40)	2015	0.7841	0.9701	0.9578
Cross modality learning (17)	2016	0.7507	0.9793	0.9581
RU-Net and R2U-Net (9)	2018	0.7756	0.9820	0.9634
U-Net+joint losses (23)	2018	0.7633	0.9809	0.9610
LadderNet (22)	2018	0.7978	0.9818	0.9656
U-Net+joint losses (23)	2018	0.7633	0.9809	0.9610
Three-stage DL Model (25)	2019	0.7641	0.9806	0.9607
GNN (41)	2019	0.9463	0.9364	0.9373
MCP-EM (42)	2019	0.8106	0.9807	0.9654
Ipn-v2 and octa-500 (27)	2019	0.8155	0.9725	0.9610
AA-UNet (34)	2020	0.8167	0.9704	0.9608
HAnet (28)	2020	0.8239	0.9813	0.9670
Iternet (36)	2020	0.7970	0.9823	0.9655
CTF-Net (29)	2020	0.7948	0.9842	0.9648
LAC network (38)	2021	0.7818	0.9819	0.9635
HDS-Net (30)	2020	0.8176	0.9776	0.9632
ResDo-UNet (39)	2021	0.8020	0.9794	0.9672
FPM-Net (proposed)	2022	0.8219	0.9840	0.9728

**Table 3C:** The comparison of the STARE data set's segmentation results using various segmentation techniques.

**Method**	**Year**	**S_e_**	**S_p_**	**A_cc_**
ECB method (43)	2012	0.7548	0.9763	0.9543
SP model (19)	2016	0.7867	0.9754	0.9566
CRF model (20)	2016	0.7680	0.9738	–
Cross modality learning (17)	2016	0.7726	0.9844	0.9628
DSM-UNet (44)	2018	0.7673	0.9901	0.9712
U-Net+joint losses (23)	2018	0.7581	0.9846	0.9612
CRF-Net (45)	2018	0.7543	0.9814	0.9632
SD-UNet (32)	2019	0.7548	0.9899	0.9725
Three-stage DL Model (25)	2019	0.7735	0.9857	0.9638
Ipn-v2 and octa-500 (27)	2019	0.7595	0.9878	0.9641
AA-UNet (34)	2020	0.7598	0.9878	0.9640
Iternet (36)	2020	0.7715	0.9886	0.9701
NFN+ Net (46)	2020	0.7963	0.9863	0.9672
D-GaussianNet (47)	2021	0.7904	0.9843	0.9837
HDS-Net (30)	2021	0.7946	0.9821	0.9626
ResDo-UNet (39)	2021	0.7963	0.9792	0.9567
FPM-Net (proposed)	2022	0.8618	0.9819	0.9727

### 4.6. Visual outcomes of suggested FPM-Net

In this instance, the suggested method's graphical outcomes for the identification of retinal vessels on the datasets of fundus image e.g., DRIVE, CHASE-DB1, and STARE are shown. (i) input original image, (ii) expert annotation (Ground truth), and (iii) FPM-Net mask are shown in Figures 5A–C.

## 5. Discussion

Precise retinal image analysis is necessary for early ophthalmic diagnosis. The complicated nature of the retinal blood vessels makes them essential biomarkers for diagnosing and analyzing many retinal disorders. However, it can be difficult to detect little changes in retinal vessels. Ophthalmologists assess and record changes in the retinal vasculature manually. To evaluate these retinal disorders through image investigation for premature diagnosis, computer vision has immense potential. Ophthalmologists and other medical professionals are dealing with a variety of diagnostic challenges with the use of deep learning techniques like medical image segmentation. Semantic segmentation using deep learning is a cutting-edge technology for medical image segmentation that helps to avoid the manual processing of images for disease or symptom diagnosis. With the advancement of supervised learning, autonomous sickness analysis is becoming more prevalent to help doctors make a quicker and more precise diagnosis. This semantic segmentation technique using deep learning will help ophthalmologists in this regard. The suggested study suggests the deep-learning-based semantic segmentation technique called FPM-Net for the detection of precise retinal vasculature in fundus images. Here, we use multiple convolution layers with a combination of depth-wise separable convolutions to lessen the overall trainable parameters. Due to the spatial information being lost as a result of the pooling of layers, we employed feature-preserving blocks to maintain feature map sizes that were large enough to handle the lost spatial information. The dense connection prevents the vanishing gradient issue that plagues traditional networks' feature latency (9), leading to improved training. This feature preserves block outcomes in improved sensitivity of the suggested FPM-Net deprived of using costly preprocessing techniques. Finally, preserved features, low-level input image information, and up-sampled spatial features are aggregated at the final concatenation stage for improved prediction accuracy. In previous studies, researchers used different networks such as AA-UNet (34), Iternet (36), NFN+ Net (46), D-GaussianNet (47), HDS-Net (30), and ResDo-Net (39) for the identification of Sensitivity (SE), Specificity (SP), Accuracy (Acc), and area under curve AUC, to measure the performance of segmentation which are frequently utilized to assess how well retinal images are segmented. But in this paper, our proposed FPM-Net produced more accurate results for SE, SP, Acc, and AUC than the rest of the research done by others. In this paper, a solid architecture is shown that enables precise semantic segmentation of the retinal blood vessels. The central ideas are discussed below.

- An efficient semantic segmentation network may give precise vessel detection deprived of the need for costly preprocessing.
- The network can learn adequate features for enhanced segmentation and quicker convergence because it delivers enhanced spatial information from the initial layers.
- Creating a shallow architecture can save many trainable parameters and it is not necessary to make feature up-sampling and feature down-sampling blocks identical. To reduce the network cost, we use depth-wise separable convolution on the deeper side of the network.
- While considering vessel segmentation, a shallower architecture with fewer layers and a smaller quantity of trainable parameters performs superior to robust architecture.
- The size of the ultimate feature map is essential. In contrast to existing architectures that significantly down-sample the image, FPM-Net avoids pooling layers and maintains enough feature map size which contains valuable features and offers better performance.
- Those techniques which are based on deep learning could help ophthalmologists do analysis more quickly and offer numerous approaches for analyzing diseases.

The original images used as input into the network, the expert-annotated image provided by experts to assess research methodologies, the predicted mask at the network's production, and the predicted mask itself are all displayed in the figures along with the suggested FPM-Net network's segmented image with the mask overlapped. Tables 3A–C describes the Numerical Comparison of the Suggested FPM-Net using the most recent method. By using our proposed method FPM-Net, there is significant improvement can be observed with DRIVE datasets, it gives S_e_, S_p_, and A_cc_ as 0.8285, 0.98270, 0.92920, for CHASE-DB1 dataset 0.8219, 0.9840, 0.9728 and STARE datasets it produces 0.8618, 0.9819 and 0.9727 respectively. Which is a remarkable difference and enhancement in results as compared to old and conventional methods.

### 5.1. Limitations and future work

Even though the suggested FPM-Net recognizes retinal vessels with better segmentation performance, the suggested technique still has certain limitations. A learning-based segmentation technique, the suggested FPM-Net largely depends on the input training data. Medical data for disease analysis are extremely challenging to organize in large quantities. The amount of training data must thus be artificially increased by data augmentation. Additionally, the learning-based approaches produce output masks depending on the knowledge they have acquired, and the network's ultimate prediction may contain pixels that are both false positive and false negative.

We want to minimize the network's overall cost in the future by efficiently reducing the number of convolutions. The proposed technique is based on deep learning, as well as its efficiency solely depends on excellent training with sufficient training data. Additionally, the accuracy of the labeling generated by an ophthalmologist directly affects the precision of learning-based techniques. This will make it feasible to evaluate how well-upcoming deep-learning techniques screen for these particular disorders. We also want to develop a little system for mobile applications that run instantly. The medical sector will subsequently utilize these networks for more semantic segmentation purposes.

## 6. Conclusion

The goal of this study was to develop a network for segmenting shallow vessels that might effectively be used to support computer-aided diagnostics in the identification and diagnosis of retinal disease. The proposed method utilized the FPM-Net shallow network, which provides a successful remedy for retinal vasculature for computer-aided diagnostics. The recommended FPM network uses less memory, has more trainable parameters, and fewer layers, and can be trained with larger mini-batch sizes. A separate network with the name of FPM-Net is used to maintain a reduced final feature map during its convolutional phase. FPM-Net contains an improved portion of FPB that incorporates an external path that saves and delivers essential spatial information to increase the accuracy and robustness of the technique. As a result, when compared to other traditional approaches for detecting retinal vessels, our suggested vessel segmentation networks are more reliable and perform better without preprocessing, and they may be utilized to help medical professionals to diagnose and analyze diseases.

## Data availability statement

Publicly available datasets were analyzed in this study. This data can be found at: Khawaja et al. (2) and Sundaram et al. (10).

## Author contributions

SI: methodology and writing—original draft. MS, MH, and AH: validations. RN and SL: supervision and writing—review and editing. All authors have read and agreed to the published version of the manuscript.
